# Plasmonic semiconductor nanogroove array enhanced broad spectral band millimetre and terahertz wave detection

**DOI:** 10.1038/s41377-021-00505-w

**Published:** 2021-03-15

**Authors:** Jinchao Tong, Fei Suo, Tianning Zhang, Zhiming Huang, Junhao Chu, Dao Hua Zhang

**Affiliations:** 1 School of Electrical and Electronic Engineering, Nanyang Technological University, Nanyang Avenue, 639798 Singapore, Singapore; 2 State Key Laboratory of Infrared Physics, Shanghai Institute of Technical Physics, Chinese Academy of Sciences, 500 Yu Tian Road, 200083 Shanghai, China

**Keywords:** Optics and photonics, Electronics, photonics and device physics

## Abstract

High-performance uncooled millimetre and terahertz wave detectors are required as a building block for a wide range of applications. The state-of-the-art technologies, however, are plagued by low sensitivity, narrow spectral bandwidth, and complicated architecture. Here, we report semiconductor surface plasmon enhanced high-performance broadband millimetre and terahertz wave detectors which are based on nanogroove InSb array epitaxially grown on GaAs substrate for room temperature operation. By making a nanogroove array in the grown InSb layer, strong millimetre and terahertz wave surface plasmon polaritons can be generated at the InSb–air interfaces, which results in significant improvement in detecting performance. A noise equivalent power (NEP) of 2.2 × 10^−14^ W Hz^−1/2^ or a detectivity (D^*^) of 2.7 × 10^12^ cm Hz^1/2^ W^−1^ at 1.75 mm (0.171 THz) is achieved at room temperature. By lowering the temperature to the thermoelectric cooling available 200 K, the corresponding NEP and D^*^ of the nanogroove device can be improved to 3.8 × 10^−15^ W Hz^−1/2^ and 1.6 × 10^13^ cm Hz^1/2^ W^−1^, respectively. In addition, such a single device can perform broad spectral band detection from 0.9 mm (0.330 THz) to 9.4 mm (0.032 THz). Fast responses of 3.5 µs and 780 ns are achieved at room temperature and 200 K, respectively. Such high-performance millimetre and terahertz wave photodetectors are useful for wide applications such as high capacity communications, walk-through security, biological diagnosis, spectroscopy, and remote sensing. In addition, the integration of plasmonic semiconductor nanostructures paves a way for realizing high performance and multifunctional long-wavelength optoelectrical devices.

## Introduction

Millimetre and terahertz wave detectors have a wide range of applications in areas such as communications, security, biological diagnosis, spectroscopy, and remote sensing^1–8^. They are the components that can transform light information loaded by long-wavelength millimetre and terahertz waves into electrical signals. High-performance room temperature detectors with high sensitivity, fast response, broad spectral bandwidth, and possibility to be extended to large format arrays are always pursued. They are the building blocks for a wide range of millimetre and terahertz wave related systems, including communication network, deep space exploration equipment, security screening system, spectroscopy system, and material composition inspection. However, conventional efficient photoexcitation in optoelectronic semiconductors seems not applicable due to small quantum energy of millimetre and terahertz waves (1.24 meV@0.3 THz) and strong background thermal disturbances (25.7 meV@300 K). Although Golay cells (such as products from Tydex and Microtech Instr.), pyroelectrics (such as DLATGS, LiTaO_3_, and products from QMC Instr.), bolometers (such as HgCdTe, SiGe, Ti, NbN, VO_*x*_, Nb, and Al/Nb), and Schottky barrier diodes (SBDs) (such as InGaAs, Er/InGaAlAs, and products from Virginia Diodes, Inc.) are in widespread use, they suffer from poor noise equivalent power (NEP) (only 10^−9^–10^−10^ W Hz^−1/2^ level for Golay cells and pyroelectrics), slow response (ms level for Golay cells, pyroelectrics and uncooled semiconducting microbolometers), or narrow spectral bandwidth (multiple modules for SBDs to achieve broad spectral bandwidth)^9^.

In the past decades, much effort has been devoted to developing novel techniques for room temperature millimetre and terahertz wave direct detection. Complementary metal–oxide–semiconductor and plasma-based field electric transistor (FET), high-electron-mobility transistor and metal–oxide–semiconductor field-effect transistor have experienced a development boom, including adoption of Si, SiGe, GaAs/AlGaAs, InGaAs, InGaP/InGaAs/GaAs, and GaN/AlGaN material systems^10,11^. These devices possess multiple electrodes and sub-micrometer scale channels to arouse photoresponse. Terahertz photoconductive antennas (PCs) are equipped with a local femtosecond laser to pump a high-resistivity and ultrashort-carrier-lifetime semiconductor (such as GaAs, InGaAs, InGa(Al)As, InP, and GaAs nanowires)^12–18^. Such technique is well adopted in terahertz time domain systems. Millimetre and terahertz wave detectors based on emerging materials^19–33^ such as black phosphorus, perovskites, carbon nanotubes, Dirac semimetals, topological insulators, particularly graphene, have also been demonstrated with plasmonic, hot electron, and photothermoelectric strategies and an NEP level of 10^−11^ W Hz^−1/2^ has been achieved^34^.

Millimetre and terahertz wave detection can also be realized based on nonequilibrium electrons induced by surface plasmon polaritons (SPPs)^35^. In this methodology, a high electron mobility plasmonic semiconductor, which possesses negative permittivity in millimetre and terahertz waves, is used in an antenna-assisted subwavelength ohmic metal–semiconductor–metal structure. Under illumination, the SPPs in such semiconductor would induce nonequilibrium electrons which form photocurrent/photovoltage under a voltage bias. In the previous work, the detection was verified by simulation and experiment from devices made of plasmonic InSb in millimetre and terahertz wave range. However, as InSb film was polished from bulk wafer and adhered to sapphire substrate, it is difficult for fabricating large format arrays. In addition, since the antenna is based on narrowband half-wave dipole antenna, it has limited coupling efficiency and is not suitable for broad spectral band detection.

Subwavelength nanostructures have demonstrated their excellent capability in controlling and modulating light–matter interactions^36–40^. However, current applications are mainly based on metals that possess plasma frequencies in visible or ultraviolet ranges of the electromagnetic spectrum. In millimetre and terahertz wave range, it is quite difficult to directly drive a strong light–matter interaction by using metallic nanostructures. Spoof SPPs from periodic holes in a metal surface is a choice to solve this issue^41,42^. Besides, owing to the relatively lower plasma frequency of the doped or narrow band gap semiconductors, they can be used as excellent plasmonic materials for longer-wavelength millimetre and terahertz waves^43–50^. The adoption of nanostructures based on plasmonic semiconductor in longer wavelength is of potential to boost multiple functionalities, for example, the photoelectrical conversion capability.

Here, we report direct detection of millimetre and terahertz waves based on epitaxially grown InSb/AlInSb/GaSb/GaAs by molecular-beam epitaxy (MBE). The InSb films in such a novel structure possess high electron mobility and negative permittivity in a broad millimetre and terahertz wave band, and further, it is suitable for fabrication of large format arrays. A broad spectral bandwidth planar equiangular spiral antenna is designed to efficiently couple millimetre and terahertz waves. A nanogroove array is fabricated in InSb layer, which can arouse strong excitation of millimetre and terahertz wave SPPs, especially at the InSb–air interfaces, leading to a general improvement of 50–100% for detection performance. An NEP of 2.2 × 10^−14^ W Hz^−1/2^ or a detectivity (D^*^) of 2.7 × 10^12^ cm Hz^1/2^ W^−1^ is achieved at 1.75 mm (0.171 THz) at room temperature. The device also shows a broad spectral band detection from 0.9 mm (0.330 THz) to 9.4 mm (0.032 THz) and a fast response speed of 3.5 µs. By moderately decreasing the temperature to the thermoelectric cooling of 200 K, the corresponding NEP, D^*^, and response speed can be further improved to 3.8 × 10^−15^ W Hz^−1/2^, 1.6 × 10^13^ cm Hz^1/2^ W^−1^, and 780 ns, respectively.

## Results

**Fig. 1:**
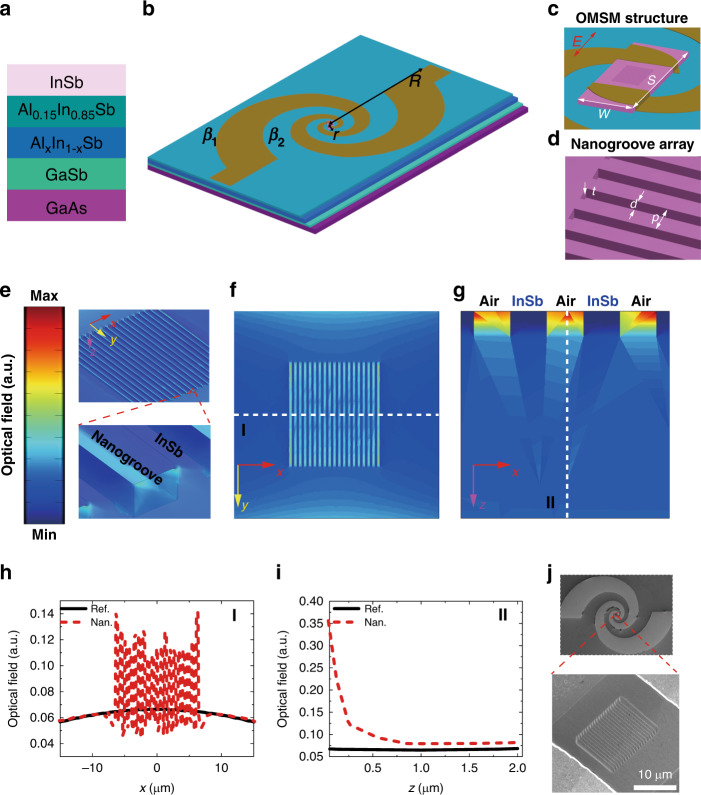
InSb device design. **a** Structure of the epitaxially grown InSb on GaAs substrate. **b** Schematic of the spiral antenna-assisted device. *R* and *r* are respectively the out and inner radius of the antenna. *β*_1_ and *β*_2_ represent curves of the arm. **c** The central ohmic metal–semiconductor–metal (OMSM structure). *s* (50 µm) and *w* (30 µm) are the length and width of the mesa between ohmic contacts. *E* denotes the TM polarization orientation (to arouse SPPs in nanogroove array) of incident electromagnetic waves. **d** Nanogroove array. The period *p*, width *d*, and depth *t* of the nanogroove are 700, 350, and 250 nm, respectively. **e** Distribution of simulated optical field for nanogroove InSb device at 0.171 THz. **f** Distribution of the field at *xy* plane (*z* = 250 nm, bottom plane of the nanogroove array). **g** Distribution of the field at *xz* (*y* = 0) plane. Optical field distribution along cut-line I (**h)** and cut-line II (**i**) as denoted in **f** and **g**, respectively. **j** Scanning electron microscopy image of the plasmonic nanogroove InSb device. The bottom panel is the zoom-in view of the nanogroove array area.

Figure 1a shows the epitaxially grown InSb-based structure on GaAs used for making devices. It contains a GaSb buffer layer (500 nm), an Al_1−*x*_In_*x*_Sb graded layer (1000 nm), an Al_0.15_In_0.85_Sb barrier layer (500 nm) and an InSb active layer (2000 nm) which are sequentially grown on a GaAs substrate (300 µm) by MBE. The measured room temperature electron concentration and mobility are 2.52 × 10^16^ cm^−3^ and 4.95 × 10^4^ cm^2^ V^−1^ s^−1^, respectively (Figs. S1 and S2). The permittivity of the InSb film in millimetre and terahertz wave range is negative in frequency up to ~3 THz or in wavelength down to 0.1 mm at room temperature according to Drude model (see “Materials and methods” and Fig. S3), which makes InSb is a good plasmonic semiconductor^44–48^. As such, millimetre and terahertz wave SPPs can be excited in InSb, which will induce the generation of nonequilibrium electrons^35,51,52^.

To obtain broad spectral band detection, a planar equiangular spiral antenna (see Fig. 1b) is designed to couple millimetre and terahertz waves. Such antenna can be described as^53^

1 $$\beta _1 = re^{\alpha \varphi };\,\beta _2 = re^{\alpha (\varphi - \theta )}$$

where *β*_1_ and *β*_2_ represent curves of the antenna’s arm, *r* is the initial inner radius, *α* is the growth factor, and *θ* is the rotation angle for the arm. This antenna includes two arms and the other arm can be determined by the same equations rotated by an angle of 180°. In our design, *θ* = *π*/2 and *α* = 0.33. The operational lower and higher frequencies of such antenna are roughly given by

2 $$f_{{\mathrm{low}}} = \frac{c}{{\sqrt 2 \pi R\sqrt {\varepsilon _{\mathrm{r}} + 1} }};\,f_{{\mathrm{high}}} = \frac{c}{{\sqrt 2 \pi r\sqrt {\varepsilon _{\mathrm{r}} + 1} }}$$

where *R* is the out radius of the antenna, *c* is the speed of light in vacuum, and *ε*_r_ is the relative permittivity of the dielectric layer on which the antenna is deposited. In our design, *ε*_r_ is around 15 for AlInSb, *r* = 0.030 mm, and *R* = 0.560 mm. Therefore, the operational frequency limit can be derived as *f*_low_ = 0.029 THz and *f*_high_ = 0.450 THz. The broadband coupling capability of this antenna was also verified by CST software (Fig. S4). It has also demonstrated great coupling efficiency for millimetre and terahertz waves^14,53^.

A plasmonic semiconductor InSb nanogroove (or slot) array was integrated into the device (as shown in Fig. 1c, d). This kind of InSb periodic structure has been demonstrated with great capability to significantly enhance the optical field (or SPPs), especially at the InSb–dielectric interface, in millimetre and terahertz wave range due to the plasmonic nature (or negative permittivity) of InSb^44,47,54–56^. These SPPs can transfer their energies to electrons or phonons in InSb, leading to absorption of incident millimetre and terahertz waves by the nanogroove array. The strong local field enhancement would induce more nonequilibrium electrons, which results in larger photocurrent/photovoltage and leading to increase in detection sensitivity^35^. As shown in Fig. 1d, the period *p*, width *d*, and depth *t* of the nanogroove array are 700, 350, and 250 nm, respectively. As such, the wavelength (mm level) of electromagnetic wave is much longer than the period (700 nm) of this nanogroove array (*λ* » *p*). Such deep subwavelength plasmonic nanogroove is expected to offer a strong excitation of SPPs.

In our design, the overall absorption of the device is first determined by the coupling of the antenna, which can be expressed as *e*_0_ = *e*_r_·*e*_c_·*e*_d_, where, *e*_0_ is the total efficiency, e_r_ is the reflection efficiency, *e*_c_ is the conduction efficiency, and e_d_ is the dielectric efficiency. Usually, it is hard to determine the three components either from calculation or experiments^53^. Even if we can determine the exact coupling efficiency, it is only the first step in the whole absorption process for the device. After coupling, the incident millimetre and terahertz waves will generate SPPs owing to the negative permittivity of InSb. In this step, only part of incident waves can generate SPPs, which then transfer their energies to electrons in InSb and therefore induce nonequilibrium electrons to form photocurrent. In this step, both the conversion ratio from coupled incident waves to SPPs and from SPPs to nonequilibrium electrons are difficult to estimate. Besides, we need to consider the influence of free-electron absorption by the metallic antenna. All these factors make it hard to determine the transmission or absorption of the device.

To gain further insight on generation of the SPPs, we simulate the distribution of SPP intensity in reference and nanogroove array InSb devices and the results for nanogroove array InSb are presented in Fig. 1e–g. As shown in Fig. 1e, obvious field enhancement is indeed observed in nanogroove InSb array with its maximum at around the interface between InSb and Air. To have a better view, we extracted the field distribution in *xy* (*z* = 0, surface of InSb) and *xz* (*y* = 0) planes for both devices (Fig. S5). As shown, InSb mesa with nanogrooves demonstrates large intensity of SPPs compared to reference one, that is, the nanogroove device will absorb more incident photons. As the SPPs in air (or within the nanogroove above the bottom surface) do not contribute to photoconductive carriers (or nonequilibrium electrons), we plot distributions of SPPs in *xy* plane located at the bottom of the nanogroove array (*z* = 250 nm) (Fig. 1f) and in *xz* (*y* = 0) plane (only a few periods in direction of *x*-axis for better view) (Fig. 1g). The InSb mesa with nanogrooves still exhibits obvious field enhancement, particularly at the interface between InSb and air in the nanogrooves, which leads to overall enhancement in the whole InSb mesa and therefore more absorption. Figure 1h, i, respectively, plot the field distribution along cut lines I and II as denoted in Fig. 1f, g. It is observed that the distribution of SPPs is kind of uniform for reference device; however, after integration of a nanogroove array, it exhibits maximum at the nanogroove area and gradually decays into InSb. Such plasmonic behavior by InSb is very like its metallic counterparts in visible range. The field enhancement in nanogroove InSb array is supposed to improve the detection performance as more nonequilibrium electrons would be induced accompanied by more absorption of millimetre and terahertz waves. It is noted here that by varying dimension of the nanogroove, SPPs excitation would be varied^55^. In this work, we try to demonstrate the capability of such nanogroove array to improve detecting performance and do not study its size-effect on performance. In fabrication, two kinds of devices, one denoted as “Ref” without nanogroove structure, and the other one denoted as “Nan” with plasmonic nanogroove array were fabricated in the same chip. The fabrication process was discussed in detail in “Materials and methods”. The scanning electron microscopy image of a typical nanogroove array device is shown in Fig. 1j.

**Fig. 2:**
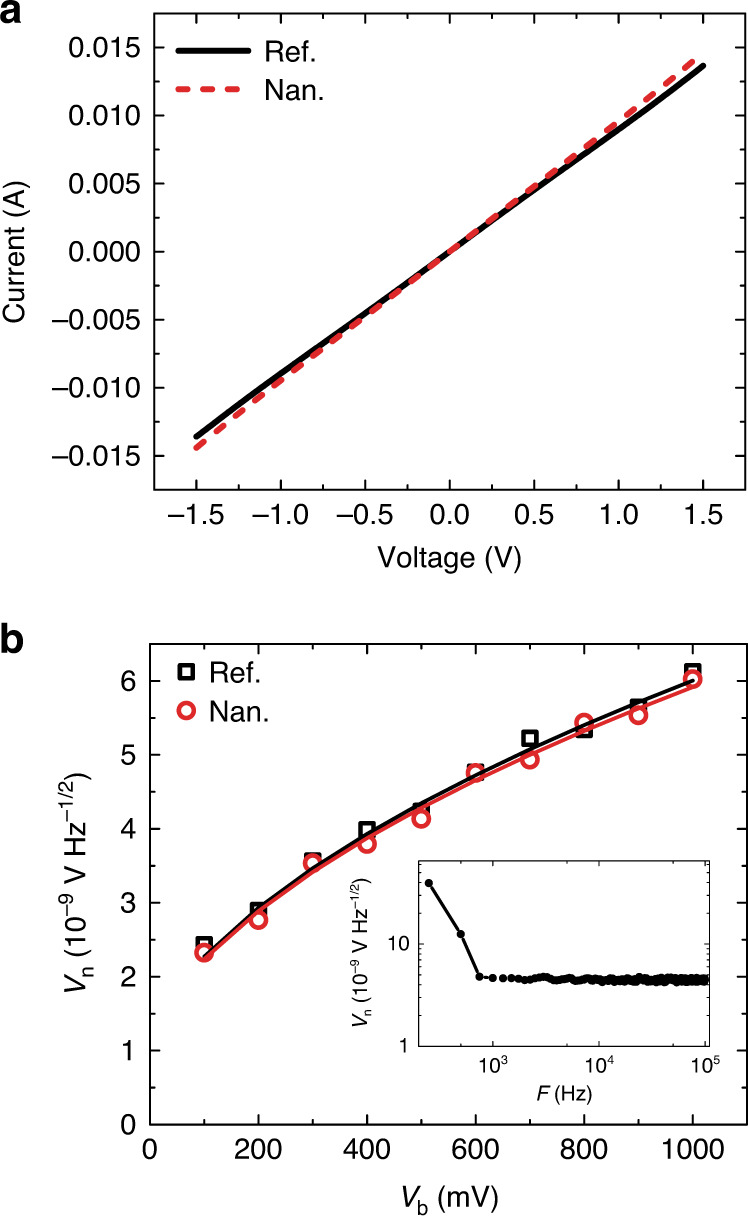
Dark current and noise characterization. **a** Current–voltage characteristic curves of reference and nanogroove devices. **b** Typical calculated (lines) and measured (squares and circles) noise level of the nanogroove and reference devices. The inset is a typical measured noise spectrum.

Dark current–voltage (*I*–*V*) curves in Fig. 2a demonstrate good ohmic and symmetry characters of devices. The nanogroove and reference devices exhibit a comparable dark current level, indicating very weak effects of the shallow plasmonic nanogroove. For our devices, in addition to thermal Johnson–Nyquist noise (*v*_t_) in FET structures, the noise (*v*_b_) due to voltage bias (dark current) should also be included. The total noise (*v*_n_) can be described by^3,19,21,27,35^

3 $$v_{\mathrm n} = (v_{\mathrm t}^2 + v_{\mathrm b}^2)^{1/2} = (4k_{\mathrm B}{\mathrm {Tr}} + 2qI_{\mathrm d}r^2)^{1/2}$$

where *k*_B_ is Boltzmann’s constant in joules per Kelvin, *T* is the detector’s absolute temperature in Kelvin, *r* is the resistance value of the device in ohms (Ω), *q* is the elementary charge, and *I*_d_ is the dark current of the device. We also used a spectrum analyzer to evaluate noise level of the devices, a typical noise spectrum can be found in inset of Fig. 2b. As shown, 1/*f* noise dominates in low-frequency range below 1000 Hz. Above 1000 Hz, the noise becomes stable at a level of 10^−9^ V Hz^1/2^. Thus, the performance characterization was conducted under a modulation frequency of 10 kHz to avoid such 1/*f* disturbance. Figure 2b plots the measured and calculated noise level of the devices under different voltage bias at 10 kHz. They agree well with each other and are comparable to other techniques at room temperature^25,57,58^. The increment at higher bias is due to the rising of dark current shot noise *v*_b_. At 1000 mV, the noise level is ~6 nV Hz^−1/2^.

**Fig. 3:**
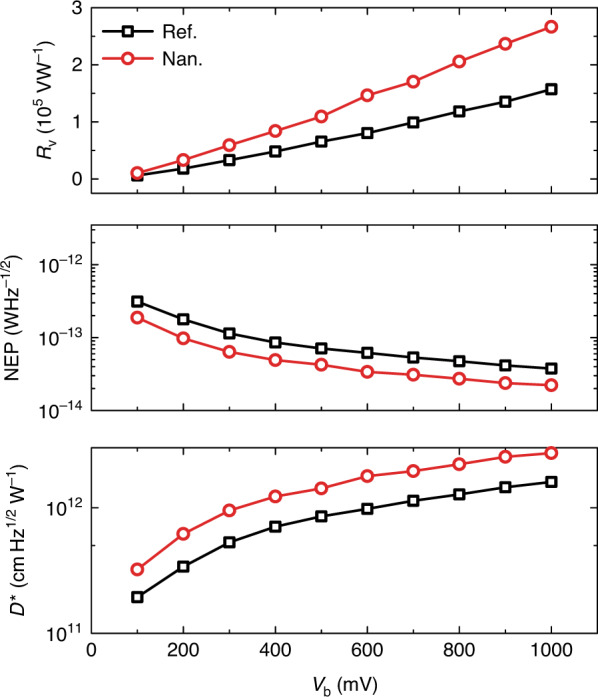
Performance vs. bias. *R*_v_, NEP, and D^*^of the devices at different voltage bias for room temperature operation at 0.171 THz.

The photoelectrical conversion capability of the devices is characterized by photoresponsivity (*R*_v_), NEP, and D^*^. They are defined as^3^

4 $$R_{\mathrm v} = V/\left( {pA} \right);\,{\mathrm {NEP}} = v_{\mathrm n}/R_{\mathrm v},\,D^ \ast = \sqrt A /{\mathrm {NEP}}$$

where *V* is the photovoltage of the device, *p* is the power density calibrated by a Golay cell and it has a typical value of 0.00171 W m^−^^2^ at 0.171 THz, *A* is the effective absorption area of the detector, described as *A* = *Gλ*^2^/(4*π*) if assuming the antenna is matched to its load (*G* is the gain of the antenna and assumed to *G* = 1 here.)^53^, and *v*_n_ is the noise voltage as described in Eq. (3). Figure 3 shows the measured *R*_v_, NEP, and D^*^ of the two devices at different voltage bias *V*_b_ for room temperature operation at 1.75 mm (0.171 THz), where devices exhibit the best performance. Performance of the nanogroove device shows an overall 50–100% improvement compared to the reference one. *R*_v_ increases linearly with voltage bias as the collected nonequilibrium electrons is proportional to the drift velocity^35^. Under a voltage bias of 1000 mV, *R*_v_ of the reference and nanogroove devices are 1.57 × 10^5^ and 2.66 × 10^5^ V W^−1^, respectively. Such high *R*_v_ is comparable to that of a Golay cell^3,9^ (1 × 10^5^ V W^−1^) and a dual-gated bilayer graphene hot electron bolometer^23^ operated at 5 K (1 × 10^5^ V W^−1^). It is much higher than other room temperature techniques^3^. For such millimetre and terahertz wave detector, due to the involvement of a couple antenna, we usually do not use quantum efficiency to evaluate its performance. For a photodiode, its quantum efficiency can be expressed as *ƞ* = (*I*_p_/*e*)/(*P*/*hv*) = (*I*_p_/*P*) × (*hv*/*e*) = *R*_A_ × (*hv*/*e*)^3,8^, where *I*_p_ is the photocurrent, *e* is the element charge, *P* is the incident power, *hv* is the quantum energy of the incident photon, and *R*_A_ is the responsivity in A/W. The typical value of *ƞ* for a photodiode is less than 100%. But for a photoconductive detector (there is no internal built-in field), its quantum efficiency is defined by the term photoconductive gain, which can be larger than 100% when expressed in percentage. Here, we can define the quantum efficiency of our detector following that of a photodiode. At 0.171 THz and 1000 mV bias, the typical quantum efficiency of our nanogroove (*R*_v_ = 2.66 × 10^5^ V W^−1^, *R* = 105.7 Ω) and reference (*R*_v_ = 1.57 × 10^5^ V W^−1^, *R* = 111.7 Ω) devices are 176% and 98% respectively, or we say, the photoconductive gain are 1.76 and 0.98, respectively. NEP and D^*^ also show improvement while increasing bias, but due to increment of noise level (see Fig. 2), they tend to saturate at a relatively large bias of 1000 mV. At this point, NEP of the reference and nanogroove devices are as low as 3.7 × 10^−14^ and 2.2 × 10^−14^ W Hz^−1/2^, respectively, demonstrating a 1–2 order of magnitude improvement compared to commercially available zero-bias SBD (10^−12^ W Hz^−1/2^ level^59^) (performance of other uncooled techniques has been summarized in refs. ^3,35^ and Table S1). The corresponding D^*^ reaches to 1.6 × 10^12^ and 2.7 × 10^12^ cm Hz^1/2^ W^−1^, which are two orders of magnitude higher than that of an ideal thermal-type detector^3^ (~1.9 × 10^10^ cm Hz^1/2^ W^−1^ level if assuming 2*π* FOV@300 K).

**Fig. 4:**
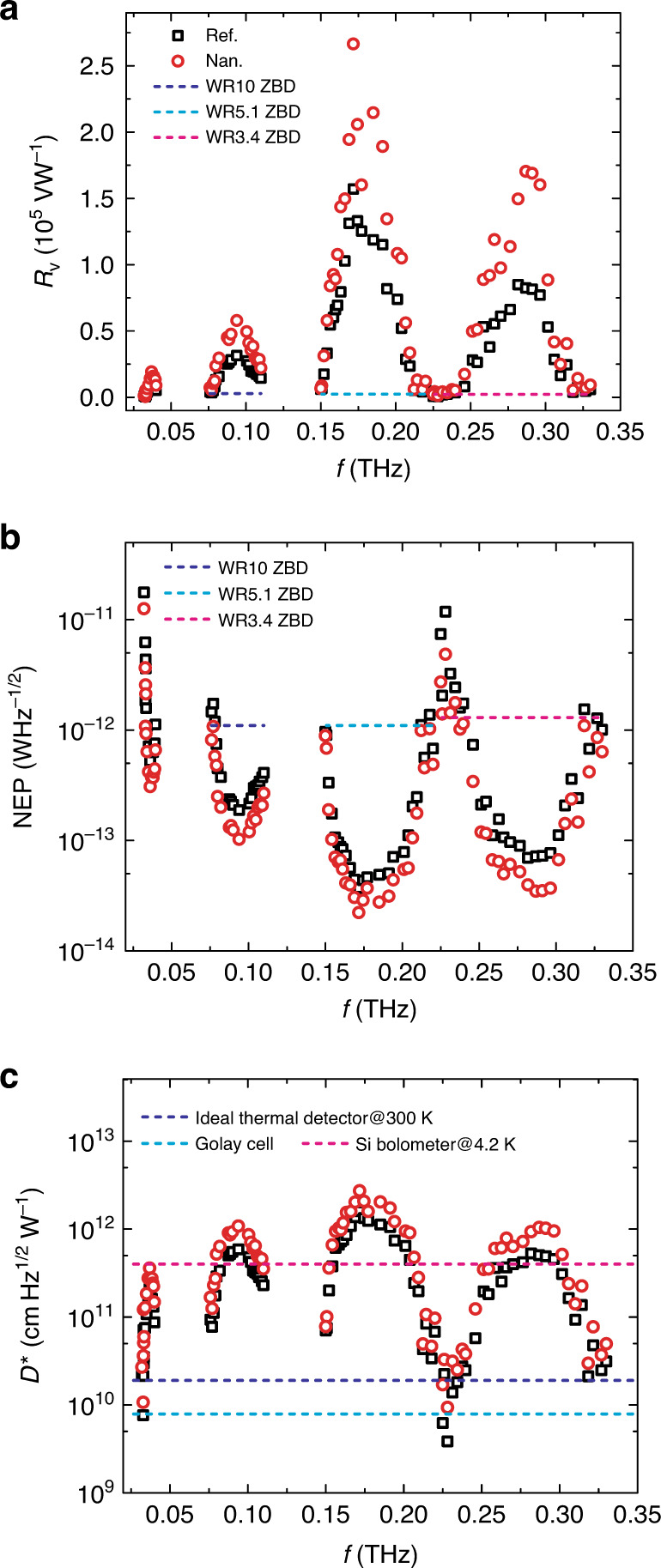
Spectral response. *R*_v_ (**a**), NEP (**b**), and D^*^ (**c**) of the devices in a frequency range of 0.032–0.330 THz under a voltage bias of 1000 mV. The dash lines in **a** and **b** represent typical performance of state-of-the-art commercial zero-bias SBDs (ZBDs) from VDI for corresponding frequency band^59^. The dash lines in **c** represent the ideal D^*^ of a thermal-type detector at room temperature, Golay cell and an Si bolometer at 4.2 K.

Spectral response was characterized in a broad frequency range from 0.032 to 0.330 THz limited by our facility (Fig. 4). The corresponding quantum efficiency is also derived as shown in Fig. S6. The experimental process is discussed in greater detail in “Materials and methods”. Both devices demonstrate good photoresponse in four frequency bands (0.03–0.04, 0.075–0.110, 0.150–0.220, and 0.225–0.330 THz). Such broad spectral bandwidth nature can be partially ascribed to the broadband coupling capability of the spiral antenna^14,53^, which is much more efficient than the narrowband dipole-like antenna adopted in the previous report^35^. In addition, negative permittivity of InSb over a broad millimetre and terahertz wave band (up to ~3 THz) allows efficient generation of localized SPPs (Fig. S3), and therefore, broad spectral bandwidth photoresponse. The multiple peak response character is caused by the multiple internal reflection from the substrate interface (or excitation of substrate modes), which can lead to antenna pattern (gain and input impedance) degradation^60^. This phenomenon can be solved by making substrate thinner, using interconnection metallization, integrating a substrate lenses, or using a conductive substrate^61–63^. The difference for peak performance in the four bands may originate from the difference in coupling efficiency of the antenna. As shown in Fig. S4, the antenna can couple the maximum optical field (largest power intensity) into the device at 0.171 THz, then sequentially decreased at 0.287, 0.094, and 0.037 THz. It may also be related to conversion efficiency from coupled light to SPPs and from SPPs to nonequilibrium electrons.

In Fig. 4a, b, we plot the typical *R*_v_ (~2000 V W^−1^) and NEP (~1 × 10^−12^ W Hz^−1/2^) of state-of-the-art zero-bias diodes (ZBDs) in corresponding frequency bands (ZBD module is not available for 0.03–0.04 THz bands and D^*^ is not used as a figure of merit for ZBDs). As shown, our devices demonstrate a general 1–2 orders of magnitude improvement in performance in a broadband range. With a single device, we achieve broad spectral bandwidth detection, which, in contrast, requires multiple modules for ZBD^57^ (WR10, WR5.1, and WR3.4 as denoted in Fig. 4). In Fig. 4c, we plot D^*^ of an ideal thermal detector^3^, a Golay cell (room temperature operation), and an Si bolometer (4.2 K). Our nanogroove device exhibits a 1–2 orders of magnitude improvement compared to Golay cell. D^*^ is also higher than that of an ideal thermal-type detector at room temperature and it is even higher than that of the Si bolometer (4.2 K) in the frequency bands of 0.080–0.100, 0.155–0.204, and 0.266–0.300 THz. Specifically, at 0.287 THz (1.05 mm), *R*_v_ of our reference and nanogroove devices are respectively 8.2 × 10^4^ and 1.7 × 10^5^ V W^−1^. The measured NEP are 7.2 × 10^−14^ and 3.5 × 10^−14^ W Hz^−1/2^, respectively. D^*^ still shows a level of 10^12^ cm Hz^1/2^ W^−1^ for both. The NEPs in this frequency band are comparable to that of the plasmonic InAlAs/InGaAs/InP detector, which achieved a NEP of 4.8 × 10^–13^ W Hz^−^^1/2^ at 0.2 THz^64^. The performance decrease in low-frequency bands. For example, at 0.094 THz (3.19 mm), we achieve a *R*_v_, NEP, and D^*^ of 5.8 × 10^4^ V W^−1^, 1.0 × 10^−13^ W Hz^−1/2^, and 1.1 × 10^12^ cm Hz^1/2^ W^−1^, respectively, which is comparable to the InAs/AlSb/AlGaSb heterojunction backward diode (NEP = 1.8 × 10^−13^ W Hz^−1/2^) at the same frequency^65^ and the AlGaN/GaN high electron mobility transistor at 0.14 THz (5.8 × 10^–13^ W Hz^−^^1/2^)^66^. Even at 0.037 THz (8.11 mm), the *R*_v_, NEP, and D^*^ of the nanogroove device can reach to 1.9 × 10^4^ V W^−1^, 3.1 × 10^−13^ W Hz^−1/2^, and 3.6 × 10^11^ cm Hz^1/2^ W^−1^, respectively. It is noted here that as the negative permittivity of InSb can hold until ~3 THz (Fig. S3), our InSb devices are promising for even higher frequency detection by engineering the coupling antenna, for example, by reducing the inner radius.

**Fig. 5:**
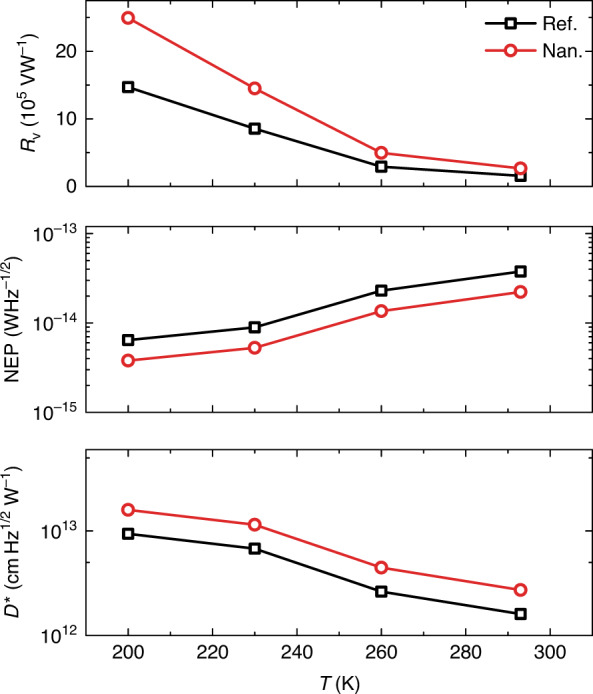
Performance vs. temperature. *R*_v_, NEP, and D^*^ of the devices at different temperature under a voltage bias of 1000 mV at 0.171 THz.

Temperature dependence of performance was characterized from room temperature (293 K) to thermoelectric cooling available up to 200 K (Fig. 5). As shown, the performance shows an overall improvement while decreasing the temperature. At 200 K, *R*_v_, NEP, and D^*^ of the nanogroove device are 2.5 × 10^6^ V W^−1^, 3.8 × 10^−15^ W Hz^−1/2^, and 1.6 × 10^13^ cm Hz^1/2^ W^−1^, respectively, demonstrating a 5–6-fold enhancement compared to those at room temperature. Such performance improvement can be mainly ascribed to the increase of electron mobility at low temperatures^35,67^ (Fig. S2). We do not study the performance of our devices at extremely low temperatures as our interests lay on uncooled or cheap thermoelectric cooling technology-based applications.

**Fig. 6:**
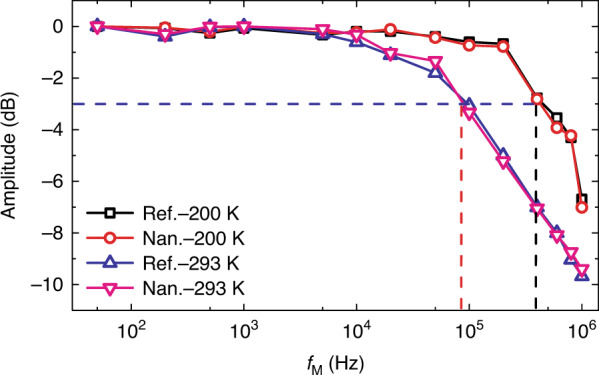
Response bandwidth. Amplitude–frequency response of the devices at 293 and 200 K.

To gain an insight on response speed, we measured photoresponse of devices with respect to modulation rate of incident wave at 0.171 THz under a bias of 1000 mV (Fig. 6). Both devices demonstrate a 3 dB bandwidth of 10^5^ Hz for room temperature operation, corresponding to a rise time (signal from 0 to 63.2 of the maxima) of 3.5 µs according to *t*_r_ = 0.35/*f*_−__3dB_, which is much faster than commercial Golay, pyroelectric and uncooled semiconducting microbolometer detectors (ms level)^5^. It is slower than metallic microbolometers^68–71^, which possess a response speed faster than ~3 µs. It is also much slower than that of ZBDs, which can respond as fast as nanosecond level (GHz bandwidth)^59^. Nevertheless, it can still meet the requirement of real-time imaging applicaions. At 200 K, the corresponding 3 dB bandwidth and rise time are 4.5 × 10^5^ Hz and 780 ns, respectively, owing to the fast transition of nonequilibrium electrons at low temperature. In experiments, we observed very close response speed at other radiation frequencies. These devices operate on single type carrier mode (electrons). The detection is based on the undirectional flow of nonequilibrium electrons induced by SPPs. The lifetime of the nonequilibrium electrons here is similar to that of hot electrons in bulk InSb, which is around 50 ns at room temperature^72,73^. The response speed of our devices are probably governed by the decay time of SPPs, the lifetime of those nonequilibrium electrons, the time for photoconductive carriers to be collected by electrodes, and the RC factor in the whole measurement set-up^35^. Further study needs to be conducted to figure this out. In experiments, we also found that the photovoltage of the device is proportional to the power of radiation. As shown in Fig. S7, the linear dynamic range is derived as 21.7 dB at 0.171 THz (limited by the maximum output power of the source).

## Discussion

In conclusion, we have demonstrated sensitive and broadband millimetre and terahertz wave photodetectors that are based on epitaxially grown InSb/AlInSb/GaSb/GaAs. A plasmonic nanogroove array is fabricated in the device to drive an overall 50–100% performance improvement. At room temperature, the device achieves a NEP of 2.2 × 10^−14^ W Hz^−1/2^ and a D^*^ of 2.7 × 10^12^ cm Hz^1/2^ W^−1^ at 1.75 mm under an applied bias of 1000 mV. The corresponding NEP and D^*^ can be further improved to 3.8 × 10^−15^ and 1.6 × 10^13^ cm Hz^1/2^ W^−1^ while operating at 200 K. The single plasmonic device achieves broad spectral bandwidth from 0.9 mm (0.330 THz) to 9.4 mm (0.032 THz). Fast response speed of 3.5 µs and 780 ns are, respectively, achieved at room temperature and 200 K. Such high-performance photodetector would be a strong building block in wide applications of millimetre and terahertz waves. The integration of plasmonic semiconductor nanostructures is promising for developing novel and high-performance millimetre and terahertz wave optoelectrical devices.

## Materials and methods

### Device simulation

In simulation, CST software was used to evaluate the coupling efficiency of the antenna. The typical coupling for four frequencies is presented in Fig. S4 (see Supporting Information). COMSOL Multiphysics was used to calculate the SPP generation in the plasmonic InSb nanogroove array. The frequency dependence of relative permittivity of InSb in millimetre and terahertz wave range can be described as *ε*(*ω*) = *ε*(0)−*ω*_p_^2^/(*ω*^2^ + *iωτ*^−1^)^45,55,56^ (*ε*(0) is the static dielectric constant and is 16.8 for InSb, *ω*_p_ is the plasma frequency, and *τ* is the relaxation time of the electrons). *ω*_p_ is determined by *ω*_p_^2^ = *q*^2^*n*/(m^*^ε_0_), where *q* is the elementary charge, *n* is the electron density, m^*^ is the electron effective mass at the bottom of the band edge and is 0.014*m*_0_ for InSb, and *ε*_0_ is the permittivity in vacuum. The mobility of the carriers is related to the relaxation time by *µ* = *qτ*/m^*^. The calculated relative permittivity of InSb in frequency of interest is presented in Fig. S3.

### Device fabrication

SiO_2_ (150 nm) was first deposited on InSb epitaxial wafer, which was then patterned with electron-beam lithography using a negative resist ZEP. The patterned features were first etched with RIE to define SiO_2_ hard mask. The wafer was then etched by ICP-RIE to define the plasmonic InSb nanogroove array after resist removal by PR Asher. Before UV lithography to define InSb mesa, residual SiO_2_ was removed by BOE solution. The mesa etch was conducted based on chemical solution (HF:H_2_O_2_:citric acid:DI H_2_O). Next, metallic contacts and antennas (300 nm Au followed by 20 nm Ti adhesion layer) were defined via photolithography, e-beam evaporation, and a standard lift-off process. Multiple reference and nanogroove devices are processed in the same chip.

### Device characterization

Dark current–voltage characteristics were measured under blackout conditions with a Keithley 2450 source meter. Noise spectra were obtained by a single channel 100 kHz SR770 FFT spectrum analyzer. Millimetre and terahertz wave sources are based on commercially available VDI tenable synthesizer (0.008–0.020 THz), WR28SGX (signal generator extension module 0.026–0.040 THz), WR10SGX-M (mini signal generator extension module 0.075–0.110 THz), WR5.1 × 2 broadband Doubler (0.150–0.220 THz), WR3.4 × 3 broadband Tripler (0.225–0.330 THz), and a PIN switch for fast modulation of output. Four horn antennas used for four millimetre and terahertz wave bands are WR10CH conical horn antenna (0.075–0.110 THz), WR5.1CH conical horn antenna (0.150–0.220 THz), WR3.4DH diagonal horn antenna (0.225–0.330 THz), and WR28CH conical horn antenna (0.026–0.040 THz). The devices were mounted in a low-temperature dewar and biased by a direct current. The photovoltage output was collected by a lock-in amplifier after a preamplifier. The output radiation power from horn antennas was calibrated by a thermal Golay cell. The typical power density of the source at 0.171 THz is 0.00171 W m^−2^.

## Supplementary information

Supplementary Information for Plasmonic semiconductor nanogroove array enhanced broad spectral band millimetre and terahertz wave detection
