# Supplementary material for: Plasmonic semiconductor nanogroove array enhanced broad spectral band millimetre and terahertz wave detection
Source: Light Sci Appl. 2021 Mar 15;10:58. doi: 10.1038/s41377-021-00505-w (PMC7961140; doi:10.1038/s41377-021-00505-w)
Supplement: Supplementary file 1 — Supplementary Information for Plasmonic semiconductor nanogroove array enhanced broad spectral band millimetre and terahertz wave detection [file 41377_2021_505_MOESM1_ESM.docx]

Supplementary Information for

**Plasmonic semiconductor nanogroove array enhanced broad spectral band millimetre and terahertz wave detection**

Jinchao Tong^1*^, Fei Suo^1^, Tianning Zhang^1^, Zhiming Huang^2^, Junhao Chu^2^ & Dao Hua Zhang^1*^

^1^School of Electrical and Electronic Engineering, Nanyang Technological University, Nanyang Avenue, 639798, Singapore.

^2^State Key Laboratory of Infrared Physics, Shanghai Institute of Technical Physics, Chinese Academy of Sciences, 500 Yu Tian Road, 200083, Shanghai, China.

Correspondence and requests for materials should be addressed to D.H.Z. (email: edhzhang@ntu.edu.sg) and J.C.T. (email: jctong@ntu.edu.sg).

**Fig. S1 Electron concentrations of the InSb layer epitaxially grown on GaAs substrate at different temperature. At room temperature and above, the electron concentration is the intrinsic value.**

**Fig. S2 Electron mobility of the InSb layer epitaxially grown on GaAs substrate at different temperature.**

**Fig. S3 Permittivity of InSb in millimetre and terahertz wave range at room temperature.**

**Fig. S4 Coupling capability of antenna.** The coupling capability of the spiral antenna is simulated at typical frequencies in a broad band range of 0.032-0.330 THz. At each frequency, the antenna can couple incident waves into the central active InSb layer. The maximum optical field at 0.0171 THz, 0.0287 THz, 0.094 THz, and 0.037 THz are 6.5×10^5^ V m^-1^, 4.8×10^5^ V m^-1^, 1.6×10^5^ V m^-1^, and 1.3×10^5^ V m^-1^, respectively.

**Fig. S5 SPPs generation.** Optical field distribution in the reference (Ref.) and nanogroove (Nan.) array InSb at xy plane (z=0, surface of InSb) and xz (y=0) plane at 0.171 THz.

**Fig. S6** Quantum efficiency of the detectors at 1000 mV bias.

**Fig. S7** Photovoltage output of the device with respect to output power of the source at 0.171 THz. The derived linear dynamic range (LDR) is 21.7 dB in the measurement range limited by the maximum output power of the source. LDR=10·lg(P_max_/(P_min_ or NEP)

**Supplementary Table S1** Summary of the state-of-the-art uncooled millimetre and terahertz wave detectors.

| **Device** | **NEP (W Hz^-1/2^)** | **Frequency (THz)** | **Response time (s)** | **ref** |
| --- | --- | --- | --- | --- |
| Golay cells | 10^-10^-10^-9^ | 0.02-30 | 2.5-5×10^-2^ | 1,2 |
| Pyroelectric | 10^-9^ | <30 | 10^-1^ | 3 |
| Schottky diodes | 10^-12^ | 0.075-0.110 (WR10); 0.140-0.220 (WR5.1); 0.220-0.330 (WR3.4); | <10^-9^ | 4 |
| Bolometers (HgCdTe, SiGe, Ti, NbN, VO_x_, Nb and Al/Nb) | 10^-11^-10^-9^ | <3 | 10^-6^-10^-3^ | 5 |
| Si FET or Si CMOS | 10^-11^-10^-10^ | <0.7 | - | 6,7 |
| CMOS-based (SiGe, GaAs/AlGaAs, InGaAs, InGaP/InGaAs/GaAs, GaN/AlGaN) | 10^-11^-10^-9^ | <3 | - | 5 |
| Photoconductive antenna | - | 0.1-1.5 (depends on antenna) | <10^-9^ | 8,9 |
| Graphene | 3×10^-8^ | 0.3 | - | 10 |
| Graphene | 2×10^-11^ | 2.52 | <10^-9^ | 11 |
| Black Phosphorus | 4×10^-8^ | 0.3 | - | 12 |
| Polished InSb on Sapphire | 1.5×10^-13^ | 0.01-1 (depends on antenna) | 10^-6^ | 13 |
| CH_3_NH_3_PbI_3_ Perovskite | 3×10^-10^ | 2.52 | 1.26×10^-7^ | 14 |
| Suspended Carbon Nanotube Films | 1.6×10^-8^ | 2.52 | 7×10^-2^ | 15 |
| EuBiSe_3_ Single Crystal | 6.7×10^-10^ | 2.52 | 3.42×10^-1^ | 16 |
| 3D graphene FET | 4.8×10^-11^ | 3.13 | 2.65×10^-7^ | 17 |
| PtTe_2_ Dirac semimetal | 1×10^-11^ | 0.12 | 2×10^-5^ | 18 |
| InAs/AlSb/AlGaSb | 1.8×10^-13^ | 0.094 | 10^-9^ | 19 |
| AlGaN/GaN | 5.8×10^–13^ | 0.14 | - | 20 |
| InAlAs/InGaAs/InP | 4.8×10^–13^@0.2 THz | 0.2–0.292 | - | 21 |
| **InSb/AlInSb/GaSb/GaAs** | **2.2×10^-14^@0.171 THz** | **0.032-0.330** | **3.5×10^-6^** | **This work** |

**Supplementary References**

1. Golay Cells Datasheet. http://www.tydexoptics.com/pdf/Golay_Detectors.pdf. Models GC-1P/T/D. Accessed September 3, 2020.
2. Golay Cells Datasheet. http://mtinstruments.com/Golay_Cell_Data.pdf. Accessed September 3, 2020.
3. Pyroelectric detector features. http://www.terahertz.co.uk/qmc-instruments-ltd/thz-detector-systems/pyroelectric-detectors. Accessed September 3, 2020.
4. VDI ZBDs. https://www.vadiodes.com/en/products/detectors. Accessed September 3, 2020.
5. Rogalski, A. *Infrared Detectors*, 3rd edn (CRC Press, 2019).
6. Tauk, R. et al. Plasma wave detection of terahertz radiation by silicon field effects transistors: Responsivity and noise equivalent power, *Appl Phys Lett* **89**, 253511 (2006).
7. Pfeiffer,U. R & Ojefors, E. Terahertz imaging with CMOS/BiCMOS process technologies, *36^th^ European solid-state circuits Conference.* 13–17, 09 (2010).
8. Peng, K. et al, Single Nanowire Photoconductive Terahertz Detectors, *Nano Lett****.* 15**, 206−210 (2015).
9. Castro-Camus, E. et al., Polarization-sensitive terahertz detection by multicontact photoconductive receivers, *Appl. Phys. Lett.* **86**, 254102 (2005).
10. Vicarelli, L. et al., Graphene field-effect transistors as room-temperature terahertz detectors, *Nat. Mater*, **11**, 865-871 (2012).
11. Cai, X. H et al. Sensitive room-temperature terahertz detection via the photothermoelectric effect in graphene. *Nat. Nanotech.* **9**, 814-819 (2014).
12. Leonardo, V. *et al*., Black Phosphorus Terahertz Photodetectors, *Adv. Mater.* **27**, 5567–5572. 2015.
13. Tong, J. *et al.* Surface plasmon induced direct detection of long wavelength photons. *Nat. Commun.* **8,** 1660 (2017).
14. Li, Y. *et al.* Ultrabroadband, Ultraviolet to Terahertz, and High Sensitivity CH_3_NH_3_PbI_3_ Perovskite Photodetectors. *Nano Lett.* **20,** 5646–5654 (2020).
15. Liu, Y. *et al.* High-Performance, Ultra-Broadband, Ultraviolet to Terahertz Photodetectors Based on Suspended Carbon Nanotube Films. *ACS Appl. Mater. Interfaces* **10**, 36304–36311 (2018).
16. Wang, Y. *et al.* Ultrabroadband, Sensitive, and Fast Photodetection with Needle-Like EuBiSe 3 Single Crystal. *ACS Photonics* **6**, 895–903 (2019).
17. Deng, T. *et al.* Three-Dimensional Graphene Field-Effect Transistors as High-Performance Photodetectors. *Nano Lett.* **19**, 1494–1503 (2019).
18. Xu, H. *et al.* PtTe 2 ‐Based Type‐II Dirac Semimetal and Its van der Waals Heterostructure for Sensitive Room Temperature Terahertz Photodetection. *Small* **15**, 1903362 (2019).
19. Ze Zhang, Rajavel, R., Deelman, P. & Fay, P. Sub-Micron Area Heterojunction Backward Diode Millimeter-Wave Detectors With 0.18 pW/Hz^1/2^ Noise Equivalent Power. *IEEE Microw. Wirel. Components Lett.* **21**, 267–269 (2011).
20. Hou, H. W., Liu, Z., Teng, J. H., Palacios, T. & Chua, S. J. High Temperature Terahertz Detectors Realized by a GaN High Electron Mobility Transistor. *Sci. Rep.* **7**, 46664 (2017).
21. Kurita, Y. *et al.* Ultrahigh sensitive sub-terahertz detection by InP-based asymmetric dual-grating-gate high-electron-mobility transistors and their broadband characteristics. *Appl. Phys. Lett.* **104**, 251114 (2014).
